# Gender Differences in the Impact of the COVID-19 Pandemic on Employment, Unpaid Work and Well-Being in the EU

**DOI:** 10.1007/s10272-021-0994-5

**Published:** 2021-10-05

**Authors:** Sanna Nivakoski, Massimiliano Mascherini

**Affiliations:** Social Policies, Eurofound, Wyattville Road, D18 KP65 Loughlinstown, Co. Dublin, Ireland

## Abstract

Unpaid work carried out inside the home has increased in the pandemic, and evidence points to women’s share in care responsibilities and domestic tasks remaining higher than those of men in the pandemic, continuing the gender divides of past decades.

On 11 March 2020, the World Health Organization (WHO) declared COVID-19 a pandemic. Within a matter of days, Dr Tedros Adhanom Ghebreyesus, WHO Director-General said that Europe had become the epicentre of the pandemic, with more reported cases and deaths than the rest of the world (WHO, 2020).

With the continent trying desperately to contain the spread of the virus through the introduction of restrictive social measures, what initially started as a health crisis quickly developed, as a result of protracted lockdowns, into a profound economic recession and further into a social crisis that deeply affected the working and living conditions of all European citizens.

Like a modern Kerberos, the gigantic three-headed hound and guardian of Hades, the health, economic and social faces of the COVID-19 crisis affected various socio-demographic groups in our societies very differently.

Eighteen months into the COVID-19 pandemic, a total of 20,507,518 years of life have been lost due to 1,279,866 deaths from the disease in the 81 countries studied (i Arolas et al., 2021). With an average of 16 years of life lost per death, the age-specific trends in COVID-19 deaths have been clear since the beginning of the pandemic. For every 1,000 people infected who are under the age of 50, almost none will die (Mallapaty, 2020). For people in their fifties and early sixties, about five will die — more men than women. The risk then climbs steeply as the years accrue. For every 1,000 people in their mid-seventies or older who are infected, around 116 will die.

* The information and views set out in this article are those of the authors and do not necessarily reflect the official opinion of the European Commission.

**Sanna Nivakoski**, Eurofound, Dublin, Ireland.

While these stark statistics confirm the acute danger of the virus to the lives of the older population, younger generations and women were those who have been the most exposed to the economic and social consequences of the crisis that were generated by the non-pharmaceutical interventions (NPIs) put in place by national governments in an attempt to control the spread of the virus.

In particular, while women appear to be more resilient than men to COVID-19 in terms of health outcomes, NPIs affected employment in sectors where women are more likely to work, and exacerbated gender divides in domestic labour and financial fragility, all to the disadvantage of women. As the work-life conflict escalated as people teleworked for extended periods of time, mothers of small children have often borne the brunt of the impact (Eurofound, 2020).

## Past recessions and the COVID-19 crisis

In past recessions, men's paid work has traditionally decreased, while that of women has increased. Men have usually suffered losses in the labour market because they are more likely to work in industries that are commonly harder-hit in recessions – such as manufacturing and construction. Within families, women have traditionally increased their labour market participation during economic downturns, as a form of within-family insurance (Alon et al., 2021). When it comes to unpaid work, existing evidence is mixed: Khitarishvili and Kim (2014) report a decrease in women's unpaid work time in a recession in the US, while MacPhail (2017) provides evidence to the contrary from Canada. In summary, recessions have traditionally *increased gender equality*, by reducing the gaps betwen men and women in paid – and perhaps also unpaid – work.

It has been suggested that the recession caused by the COVID-19 pandemic may have different impacts on gender equality than previous recessions. Emerging evidence indicates that in many countries, women's *paid work* has fallen due to both labour demand- and supply-related factors. Labour demand has decreased because close-contact activities where women are more likely to work than men – hospitality, travel, personal care, cleaning, etc. – have been curtailed as a result of NPIs (Eurofound, 2021). Women's labour supply has been hypothesised to reduce because of their higher likelihood (influenced by traditional gender norms) to give up work when domestic demands (such as housework and childcare) increase. In addition, the higher opportunity cost of men giving up paid work results from the gender wage gap, making women more likely to exit the labour market (Alon et al., 2020).

Additionally, women's *unpaid work* may have increased in the COVID-19 pandemic if women have taken on more of the extra childcare duties imposed on parents by closures of school and childcare facilities. Also, women's unpaid work may have increased if women have taken on more food preparation tasks at home (resulting from restaurant closures and teleworking), or if they have taken on more household chores (caused by less domestic help being employed in the pandemic). As a result, it is hypothesised that the COVID-19 pandemic may have *decreased gender equality* in both paid and unpaid work.

## Gender differences in the labour market

The general trend over the past decades has been a reduction in the gender gap in employment rates (see Eurofound and EIGE, 2021). In EU27 member states for example, the gap reduced from 19 to 12 percentage points (pp) between 2000 and 2019 (see Figure 1). The gap narrowed as women's employment rate caught up with that of men: over this time period, men saw an increase from 75% to 79%, while women's increased from 56% to 67%. The US gender employment gap also declined over this time period, coinciding with the overall EU rate in 2019. In the US, both genders' employment rates declined over time, and the gender gap reduction was driven by a steeper decline in the men's employment rate (from 84% to 81%) than in the women's rate (from 70% to 69%).

The aggregate trends at the EU-level mask heterogeneity among member states. While the vast majority have seen a narrowing of the gender employment gap between 2000 and 2019, the gap has widened in four member states: Hungary, Poland, Romania and Sweden. The gap widened most drastically (by seven pp) in Romania, driven by an increase in men's employment and a decrease in women's employment. In Hungary and Poland, the gender employment gap remained relatively stable at approximately 15 pp, but with the increase in men's employment outpacing that of women's. In Sweden, the gap remained close to five pp throughout the time period. The most notable reductions in the gender employment gap took place in Ireland, Greece, Cyprus, Luxembourg, Spain and Malta, with reductions ranging from 13 pp in Ireland to 31 pp in Malta. With the exception of Malta (where women's employment growth strongly outpaced that of men's), the increasing employment among women was combined with the decreasing employment among men. As of 2019, the

Figure 1

**Gender gap in employment rate 1992-2020, selected countries**

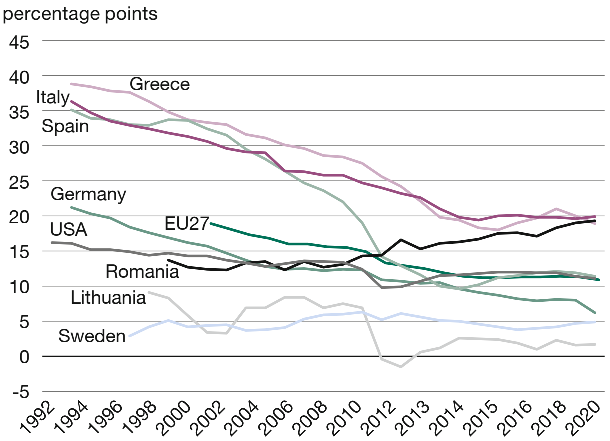

Notes: Calculated as men's employment rate minus women's employment rate; people aged 20-64.

Source: Authors' calculations based on Eurostat data. Employment and activity by sex and age (1992-2020) – annual data [LFSI_EMP_A_H].

gender employment gap was smallest (below five pp) in Latvia, Lithuania, Finland and Sweden, whereas the gap was widest (above 15 pp) in Greece, Italy, Hungary, Malta and Romania.

The early impact of the COVID-19 pandemic on gender differences in *employment* can be assessed by examining quarterly employment rates. Comparing employment figures of the second quarter of 2019 and the second quarter of 2020 (the initial shock in labour markets), the decrease over time was of similar magnitude for both men and women. A more severe impact on women can be seen in the slower recovery of women's employment between the second and third quarters of 2020 (EIGE, 2021).

Beyond the initial shock of the pandemic recession, a comparison of annual data of 2019 and 2020 suggests that overall, men's employment rate declined by more than women's – see Figure 2. Men's employment rate declined by 0.9 pp (from 79.0% to 78.1%), whereas it declined by 0.5 pp (from 67.3% to 66.8%) for women.[1] As a result, the overall EU gender employment gap reduced

1 Similarly, the change in actual weekly working hours in the main job between 2019 and 2020 aggregate annual EU27 data reveals a narrowing of the gap between men and women. The narrowing results from a reduction of 0.8 hours (from 39.0 hours to 38.2 hours) for men and a corresponding reduction of 0.5 hours (from 33.3 hours to 32.8 hours) for women (EU LFS, lfsa_ewhais). Similar findings are reported by Eurofound (2021), supplemented with evidence that women were more likely than men to have temporarily stopped working altogether.

Figure 2

**Change in gender employment gap (annual data, 2019 vs. 2020), by EU27 member state**

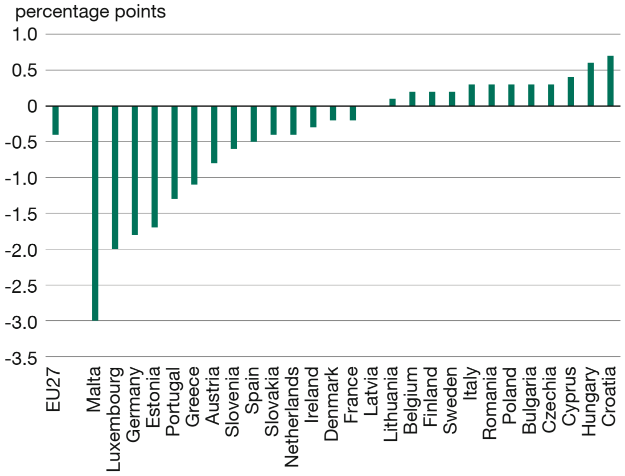

Notes: Calculated as 2020 gender employment rate gap minus 2019 gender employment rate gap. People aged 20-64.

Source: Authors' calculations based on Eurostat data. Employment and activity by sex and age – annual data [LFSI_EMP_A].

Figure 3

**Change in gender labour market inactivity rate gap (annual data, 2019 vs. 2020), by EU27 member state**

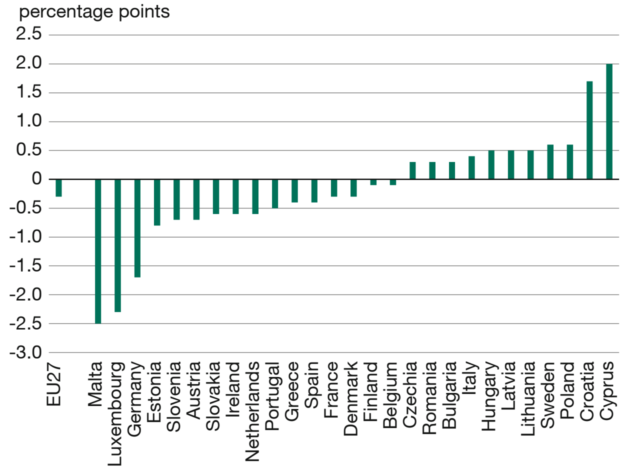

Notes: Calculated as 2020 gender labour market inactivity rate gap, minus 2019 gender labour market inactivity rate gap. People aged 20-64.

Source: Authors' calculations based on Eurostat data. Inactive population as a percentage of the total population, by sex and age (%) [LFSA_IPGA].

by 0.4 pp. Similarly, the gender employment gap narrowed in the US over the same time period. The male employment rate declined by 4.6 pp (from 80.7% to 76.1%), while it declined by 4.3 pp among women (from 69.3% to 65.0%). When it comes to differences between EU member states, Figure 2 illustrates that the apparently moderate aggregate change in the gender employment gap masked the widening of the gap in 12 member states and the narrowing of the gap in 14 member states, with the country groups largely coinciding with those reported by Alon et al. (2021). Moving beyond the EU- and country-level aggregates, the impact of the pandemic on the gender employment gap has been found to be particularly large in sectors and occupations with higher shares of women in the workforce (Eurofound, 2021).

In the EU, a gender *unemployment* gap has not been as notable in data collected before or during the pandemic (not illustrated here). Between 2019 and 2020, the annual unemployment rate increased by 0.3 pp (from 5.3% to 5.6%) among men, and by 0.2 pp (from 5.0% to 5.2%) among women.[2] In contrast, the US saw an emergence of a gender gap in unemployment, resulting from an increase in the rate by 4.1 pp among men (from 3.7% to 7.8%) but a steeper increase among women, by 4.7 pp (from 3.6% to 8.3%).[3]

2 Population aged 20-64. Authors' calculations based on Eurostat data (une_rt_a).

3 Population aged 16+. U.S. Bureau of Labor Statistics data.

Focusing on people outside of the labour market, the rate of economic *inactivity* in the EU has generally been considerably higher among women than men, and between 2019 and 2020 it increased by 0.6 pp (from 15.8% to 16.4%) among men, and by 0.3 pp (from 27.8% to 28.1%) among women, narrowing the gender gap.[4] Differences between EU member states are evident, with a widening of the gap in 11 member states and a narrowing of the gap in 16 member states, largely coinciding with the country groups emerging from the analysis of employment rates (see Figure 3).

It has been suggested that the differences between North American and European countries when it comes to the gender disparities in the labour market impacts of the pandemic are attributable to differences in protective labour market policies in response to the pandemic recession, the length of school closures and the degree of job teleworkability (Alon et al., 2021; Collins et al., 2021).

## Telework

In the pandemic, the mode of work changed drastically for large groups of workers. To curb the spread of the virus, many offices were closed and telework became the norm. The degree of teleworkability varies between sec-

4 Population aged 20-64. Authors' calculations based on Eurostat data (lfsa_ipga).

tors. Women in the EU are significantly more likely than men to work in teleworkable occupations: 45% compared to 30%, respectively (Sostero et al., 2020). As a result, 41% of women and 37% of men reported having taken up telework at the onset of the pandemic in the spring of 2020 (Sostero et al., 2020). Traditionally, an increase in telework could potentially improve work-life balance (Bloom et al., 2015; Dockery and Bawa, 2018). In a pandemic, however, telework – often compulsory as well as rapidly implemented – correlates with poor work-life balance for parents who have been juggling paid work with homeschooling and care tasks (Eurofound, 2020; Hjálmsdóttir and Bjarnadóttir, 2021).

## Gender differences in unpaid work

The adoption of NPIs meant that many activities that happened outside of the home before the pandemic were brought into the realm of home life. This transition affected many services – childcare, education, care for dependent relatives, as well as domestic work. Before the pandemic, this work was carried out outside of family units, to varying degrees. In the pandemic, as a result of closures of schools and childcare facilities, as well as the disruption of usual support networks and knock-on effects on labour supply of domestic workers, much of this activity became unpaid work that was carried out within families.

Even before the pandemic, care duties and housework have been carried out primarily by women (Eurofound, 2017). In 2016, the average weekly time spent by parents on childcare tasks was 31 hours among women and 16 hours among men.[5] With closures of schools and childcare facilities, parental time dedicated to unpaid work of this kind increased in the pandemic, and gender differences persisted. In the summer of 2020, the time spent by parents on childcare tasks averaged 37 hours per week among women and 23 hours per week among men.[6] Alon et al. (2021) suggest that, in countries with a higher rate of teleworkability of jobs, while gaps in employment between men and women may not have widened, they may have done so in unpaid work. This is because women working from home spent more time carrying out unpaid tasks.

Although the volume of childcare-related unpaid work has seen a marked increase in the pandemic, other types of unpaid work have also become more common. The average time spent on housework increased from 15 to 17 hours per week among women in the EU between 2016 and 2020; among men, the corresponding increase was from 7 to 11 hours.[7] Increases in time spent by men and women on unpaid care of relatives that are elderly or have disabilities are also evident.

Figure 4
**Risk of depression by gender and time, EU27**

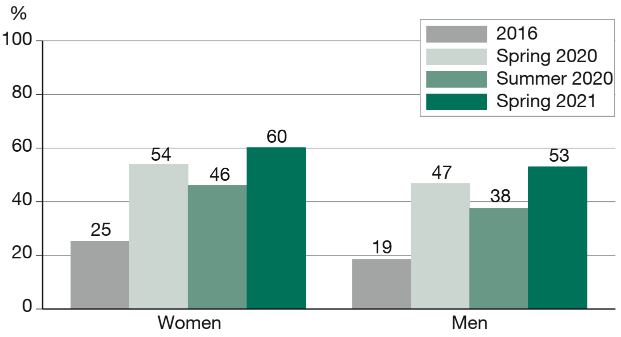

Notes: Based on WHO-5 mental well-being index. People with a WHO-5 score of 50 or lower are considered to be at risk of depression.

Sources: 2016 data from Eurofound's European Quality of Life Survey (EQLS) (EU27). 2020/2021 data form Eurofound LWC e-survey (EU27) rounds 1, 2 and 3.

## Gender differences in well-being

The widespread deterioration of mental well-being is evident in data gathered during the pandemic. In the EU, 25% of women and 19% of men were at risk of depression in 2016 (Figure 4). At the onset of the pandemic, the situation worsened markedly, with more than half (54%) of women at risk of depression in the spring of 2020. The share fell somewhat (to 46%) in the summer of 2020, but increased further (to 60%) by the spring of 2021. For men, the patterns over time have been similar to those of women, although the rates have been consistently lower: 47% of men were at risk of depression in the spring of 2020, rising to 53% a year later.

Similar to the risk of depression, financial fragility has also been more common among women in the EU before the pandemic, and the rates have increased (especially early on) in the pandemic (Figure 5). As an analysis of employment losses by job-wage quartile suggest, the pandemic has mainly affected the service sectors with a high level of social contact, including those dominated by women – where average pay levels are low (Eurofound, 2021).

5 Authors' calculations based on data from the 2016 round of Eurofound's European Quality of Life Surveys (EQLS).
6 Authors' calculations based on data from the second round of Eurofound's Living, Working and COVID-19 (LWC) e-survey.

7 Authors' calculations based on data from the 2016 round of Eurofound's European Quality of Life Surveys (EQLS) and from the second round of Eurofound's Living, Working and COVID-19 (LWC) e-survey.

Figure 5
**Financial fragility by gender and time, EU27**

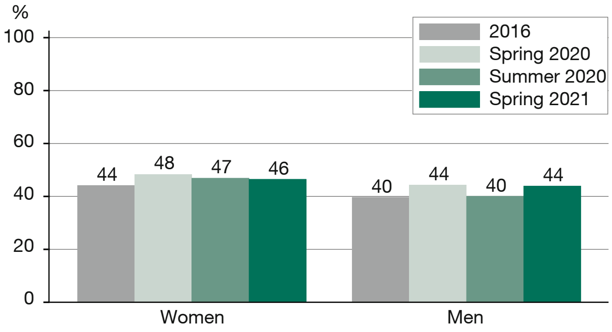

Notes: Based on the question: "A household may have different sources of income and more than one household member may contribute to it. Thinking of your household's total monthly income: is your household able to make ends meet….?" A sum of answers: "with some difficulty", "with difficulty", "with great difficulty".

Sources: 2016 data from Eurofound's European Quality of Life Survey (EQLS) (EU27). 2020/2021 data form Eurofound LWC e-survey (EU27) rounds 1, 2 and 3.

Many people have struggled to combine work and home life during the pandemic. In 2015, 20% of working women and 19% of working men reported feeling too tired after work to do household jobs (see Figure 6b). A deterioration of the situation in the pandemic is evident: in the spring of 2020, the corresponding figures were 25% and 20% among working women and men, respectively. Over the course of the pandemic, the situation deteriorated further, especially for women. As illustrated in Figure 6, family life negatively impacting work (Figures 6d and 6e) became less prevalent over the course of the pandemic, while the opposite was true for indicators of work negatively impacting family life (Figures 6b and 6c). Overall, however, work-life conflicts have remained well above pre-pandemic levels throughout the crisis.

Work-life conflicts have been especially common among working parents, especially those with younger children. In the summer of 2020, 29% of working mothers and 11% of working fathers with children under the age of 12 reported facing this problem (Eurofound, 2020).

## Conclusions

Individuals and societies worldwide have been severely impacted by the COVID-19 pandemic, but with population groups being affected in different dimensions. Concerns have been raised that some of the progress made in the past with regards to equality between men and women could be in vain if the pandemic results in a "she-cession" via a reduction in women's paid work and an increase in their share of unpaid work. While global evidence suggests that the concerns may be valid, evidence from the EU indicates that gender differences in labour market shifts have been relatively modest, with aggregate data suggesting that women have not fared worse than men when it comes to shifts in rates of employment, unemployment and economic inactivity. These averages, however, hide nuances between member states, as well as different employment sectors and occupations.

Unpaid work carried out inside the home has increased in the pandemic, and evidence points to women's share in care responsibilities and domestic tasks remaining higher than those of men in the pandemic, continuing the gender divides of past decades.

The burdens brought about by the pandemic manifest themselves in a deterioration of well-being on many fronts including the risk of depression and financial fragility, both of which have increased markedly. Women generally, and working mothers with young children particularly, have been severely impacted. This impact can also be seen in a deterioration in work-life balance.

In the longer run, the pandemic's impact on EU labour markets will become evident once crisis-related labour market policies are lifted. Longer-term shifts in labour demand (driven e.g. by shifts in consumption in sectors such as tourism and hospitality) as well as labour supply (impacted by an increase in telework and attractiveness of work in sectors such as healthcare, among other reasons) will partly shape future gender balance in labour markets. In the eventuality that women suffer more severe long-term consequences of the pandemic in the labour market, spells of unemployment or economic inactivity may result in labour market scarring, with impacts on labour market participation and career progression, as well as knock-on effects including gender gaps in pay and pensions (Alon et al., 2020).

Another factor that plays a role in the future of gender equality is the difference between men and women in the take-up of telework after the pandemic. On the one hand, teleworking may enhance flexibility, with the potential of benefitting women's employment and labour market participation in the future (Alon et al., 2021). On the other hand, if women are more likely than men to avail of teleworking, they may face worse career prospects than their male counterparts. In addition, higher teleworking among women may further increase the share of unpaid care and household work that falls on women's shoulders. Therefore, it remains to be seen what the longer-term legacy of the pandemic will be on gender equality in work and at home, once a "new normal" establishes itself.

Figure 6

**Work-life conflict by gender and time, EU27**

Based on a question "How often in the last 12 months, have you…?" / "Since you started your main paid job, how often have you…?"

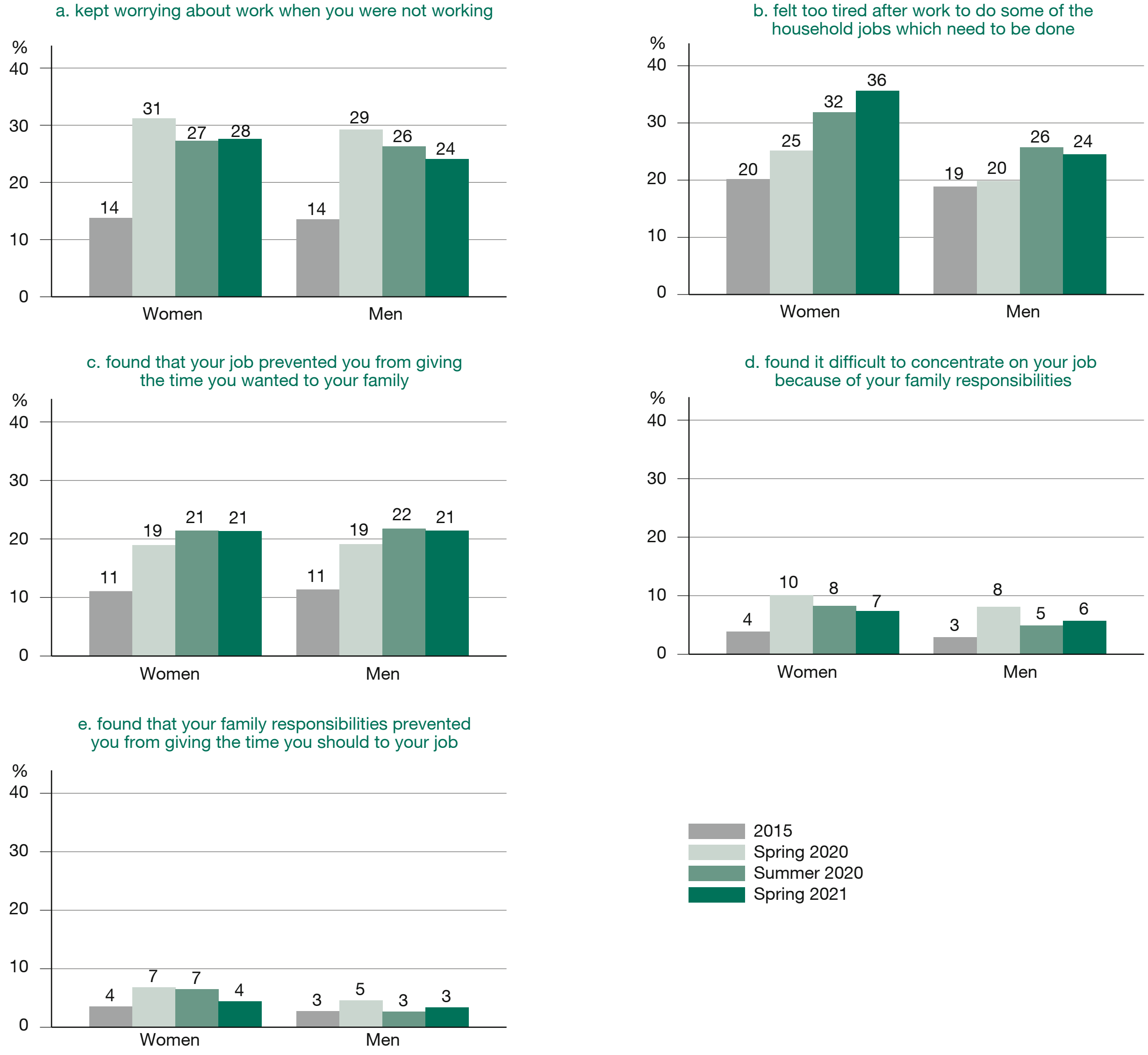

Note: A sum of answers: "always" and "most of the time"

Sources: 2015 data from Eurofound's European Working Conditions Survey (EWCS) (EU27). 2020/2021 data form Eurofound's LWC e-survey (EU27) rounds 1, 2 and 3.
